# Radiological Features of Osteoid Osteoma: Pictorial Review

**DOI:** 10.5812/kmp.iranjradiol.17351065.3392

**Published:** 2011-11-25

**Authors:** Jahanbakhsh Hashemi, Mohammad Gharahdaghi, Emad Ansaripour, Farzaneh Jedi, Sara Hashemi

**Affiliations:** 1 Department of Radiology, Imam Reza Hospital, Mashhad University of Medical Sciences, Mashhad, Iran; 2 Department of Orthopedics, Imam Reza Hospital, Mashhad University of Medical Sciences, Mashhad, Iran; 3 Department of Radiology, University Hospital Frankfurt Am Main, Johann Wolfgang Goethe University, Frankfurt Am Main, Germany; 4 Department of Pathology, Imam Reza Hospital, Mashhad University of Medical Sciences, Mashhad, Iran

**Keywords:** Osteoma, Osteoid, Magnetic Resonance Imaging, Tomography, X-Ray Computed, Radionuclide Imaging

## Abstract

Osteoid osteoma is a benign bone tumor of undetermined etiology, composed of a central zone named nidus which is an atypical bone completely enclosed within a wellvascularized stroma and a peripheral sclerotic reaction zone. There are three types of radiographic features: cortical, medullary and subperiosteal. Forty-four patients with osteoid osteoma were studied retrospectively. In plain films, 35 patients presented as the cortical type, six cases were located in the medullary zone and three had subperiosteal osteoid osteoma. In all the cases, the nidus was visualized on computed tomography (CT) scan. The nidus was visible in four out of five patients who had also undergone magnetic resonance imaging (MRI). Double-density sign, seen on radionuclide bone scans was positive in all patients. MRI is more sensitive in the diagnosis of bone marrow and soft tissue abnormalities adjacent to the lesion, and in the nidus that is located closer to the medullary zone. On the other hand, CT is more specific when it comes to detecting the lesion’s nidus.

## 1. Introduction

Osteoid osteoma was first described by Dr. Jaffe in 1935 as a benign bone tumor [1]. For several decades the orthopedic communities considered osteoid osteoma as a variant of osteomyelitis, but nowadays it has been accepted as a benign bone tumor of undetermined etiology, representing 10% of benign skeletal neoplasms [2].

Osteoid osteoma is a small spherical tumor with a diameter of 1.5 cm or less, composed of a central zone named nidus which is an atypical bone completely enclosed within a well-vascularized stroma. The peripheral sclerotic reaction zone is composed of osteoblasts, osteoclasts and dilated capillaries surrounding the nidus [3]. Peripheral nerve fibers are abundant in and around an osteoid osteoma which is a unique feature in this tumor [3]. Prostaglandins are found in the nidus at levels 100 to 1000 times that of normal tissue [4]. They induce vasodilatation and a resultant increased capillary permeability in the tissues surrounding the lesion and are believed to mediate tumor related pain, classically described as night pains relieved by salicylates. However, the inter-articular lesions have shown less response to non-steroidal anti-inflammatory drugs (NSAID_s_) compared to the extra-reticular lesions [5][6].

**Figure 1:**
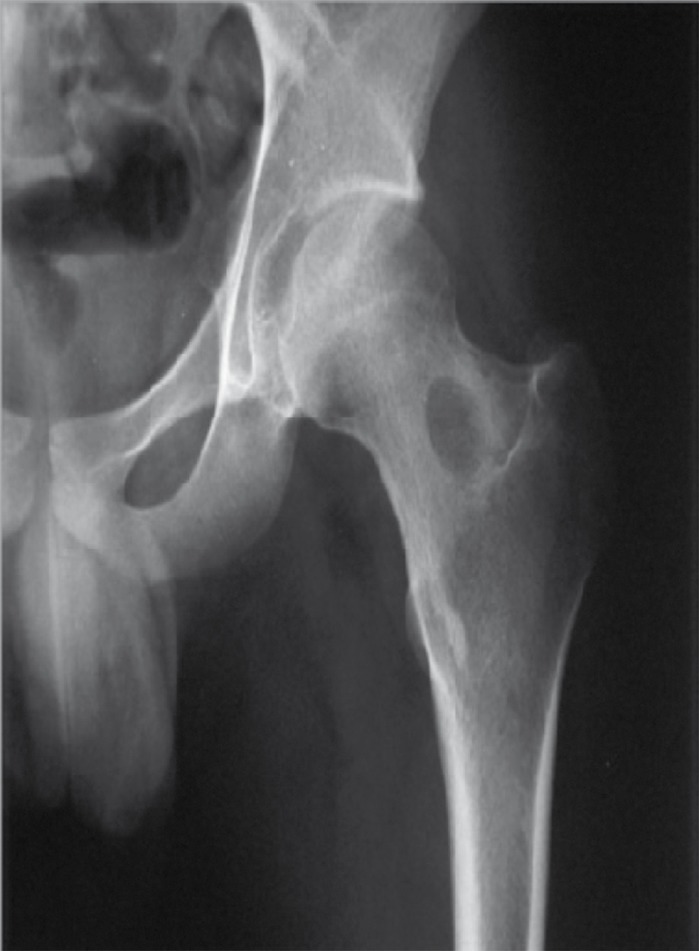
A 19-Year-Old Man With Pain in the Thigh. Body section film reveals a circular area of density with a central radiolucent nidus in the femoral neck.

This lesion can involve any bone of the skeletal system, but is mostly reported in long bones of the lower extremity; namely, the proximal femur (Figure 1) which is the origin of 25-27% of such lesions. Around 5-12% of osteoid osteomas present as inter-articular lesions [3].

**Figure 2:**
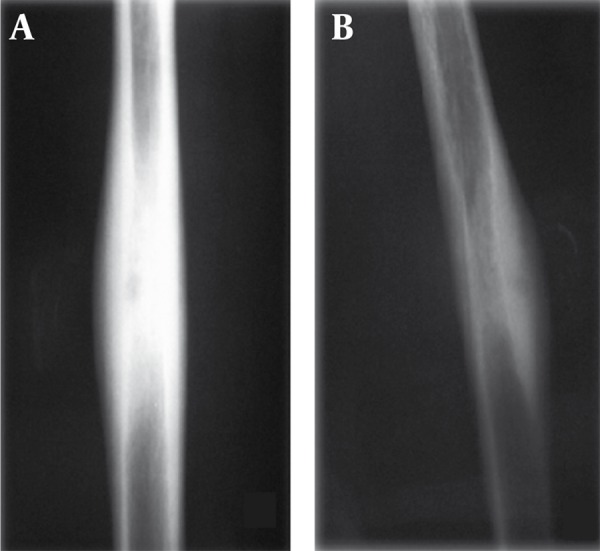
A 17-Year-Old Man With Pain in His Arm. The lesion is characterized as a small nidus surrounded by sclerotic bone. A, The nidus is not demonstrated in the AP projection but B, Precisely in the lateral projection

**Figure 3:**
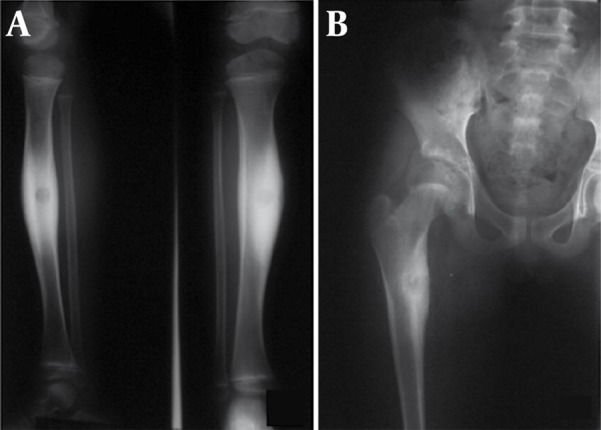
A, A 14-Year-Old Man With Leg Pain; and B, An 18-Year-Old Female With Pain in Her Thigh. In the plain film the nidus is calcified and surrounded by increased bone density.

In plain radiographs the lesion is characterized by a small nidus surrounded by dense bone (Figure 2). The nidus is mostly seen as a radiolucent area not more than 5 mm in diameter, significantly or mildly calcified depending on the disease duration (Figure 3).

Depending on the site of origin, there are three types of radiographic features; cortical, medullary and subperiosteal [1]. Cortical osteoid osteoma is the classic type of the disease consisting of a small central nidus, usually radiolucent, associated with perifocal dense bone. These lesions may appear as high density and may require overpenetrated exposures or body section techniques to visualize the nidus. The density (sclerosis) is mostly adjacent to the nidus [1].

**Figure 4:**
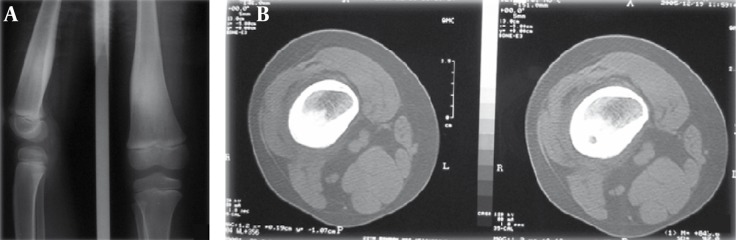
A 17-Year-Old Man Presenting With Thigh Pain. A, Plain radiographs show endosteal thickening at the distal of the femur. No definite radiolucencies are present. B, CT scan reveals the endosteal thickening and demonstrates radiolucencies in the cortex.

The medullary type involves the neck of the femur, vertebra and small bones (case 2). This type, due to formation of osteosclerosis at a distant point, is unable to cause peripheral reactive bone formation. If no reactive bone formation is present, detection of the nidus may be difficult, especially in the spinal column and femoral neck. In such a case radionuclide bone scan may be helpful [1]. The third type of osteoid osteoma is the subperiosteal type that most frequently occurs in the intra-articular portion of the bones and may be difficult to detect (case 3) consequently leading to delay in treatment [1]. Computed tomography (CT) scan is of considerable value when there is no evidence on plain films to localize the nidus of osteoid osteoma (Figure 4). CT characteristics of osteoid osteoma are:

1) A round or oval-shape low-density zone named nidus

2) High-density area within the nidus, ranging from minimal to extensive

3) Reactive peripheral sclerosis or periosteal reaction

Spinal osteoid osteoma usually occurs in the neural arch and may involve the articular process and apophyseal joints. Lesions originating from this area may be difficult to diagnose, but a painful scoliosis may be the clue to diagnosis [1]. To detect soft tissue and bone marrow abnormalities next to osteoid osteoma magnetic resonance imaging (MRI) is considered to be more sensitive than CT scan [7]. MRI is a reliable method of visualizing the nidus. The nidus may present variably in MRI depending on its relative location to the cortex. The closer the lesion is to the medullary zone, the greater the role of MRI in recognizing the nidus compared to CT scan [8].

**Figure 5:**
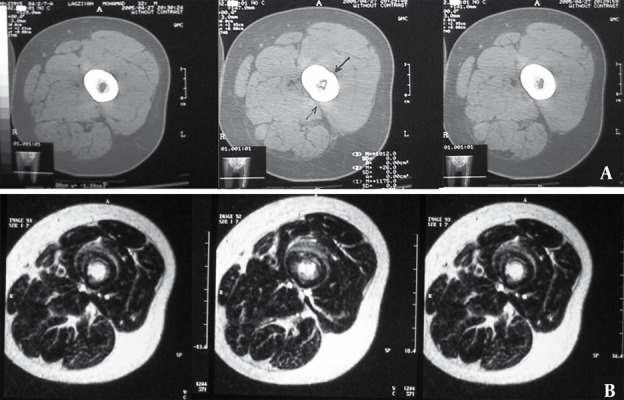
A 25-Year-Old Man Complaining of Pain in Mid FemurA. A, CT scan through mid-femur. Nidus is visible in the medulary region only in one of the cuts. B, MRI of the same patient reveals distinct hypersignal nidus in the medulary region in T2-W sequence in several cuts.

Generally, compared to MRI, CT scan is more specific for spotting a nidus. Signals in MR imaging differ among bone marrow edema, nidus and soft tissue [7]. Nonetheless, the nidus is of predominantly intermediate signal intensity on T1-weighted images and of intermediate to high signal intensity on T2- weighted images (Figure 5) [9].

**Figure 6:**
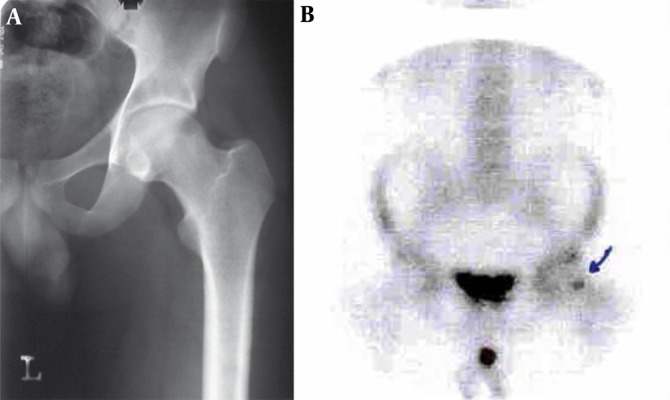
A 28-Year-Old Man Presenting With Thigh Pain. A, Plain radiograghs show no pathologic finding in cortex and medulla such as nidus or peripheral sclerotic reaction. B, Technetium scan demonstrates increased uptake in the area of the nidus (arrow). Biopsy of the mentioned lesion confirmed the diagnosis of osteoid osteoma.

If no reactive bone is apparent, the nidus may be difficult to detect and radionuclide scans are helpful in identifying the lesion (Figure 6) [1]. Spotting the double-density sign on radionuclide bone scans is diagnostic for osteoid osteoma and helps in localizing the nidus. It is also helpful to differentiate between the nidus of an osteoid osteoma and osteomyelitis [2]. We have tried to review the radiological features and clinical symptoms of this disease in 44 cases with the pathological diagnoses of osteoid osteoma.

## 2. Case Presentation

Since the aim of this assessment in which 45 patients have been reviewed is to assess and introduce different radiological features of osteoid osteoma, according to its cortical, subperiosteal or medullary bone location, we considered it sufficient to introduce five patients who had special radiological manifestations.

### 2.1. Case 1

**Figure 7:**
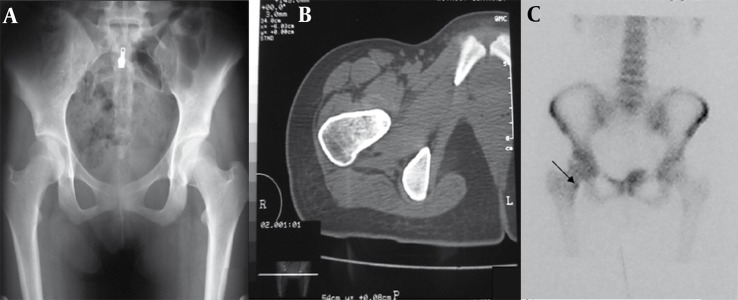
A 20-Year-Old Woman Presented With a One-Year Right Hip Pain. A, Plain radiograph of the hip revealed no abnormalities. B, CT scan of the hip shows a sclerotic lesion with a central nidus in the cortex. C, In radionuclide scan an increased uptake was detected in the same region (arrow).

A 20-year-old woman presented with right hip pain radiating to the knee from one year ago. The pain tended to worsen at night, woke her up and was relieved by asprin, but after a while the pain increased again. The plain radiograph of the hip revealed no abnormalities but CT scan showed a sclerotic lesion with a central nidus in the cortex. In radionuclide scan, an increased uptake was detected in the same region. This type of osteoid osteoma is the most common type regarding the location of the lesion (cortical) and the involved bone (femoral) (Figure 7).

### 2.2. Case 2

**Figure 8:**
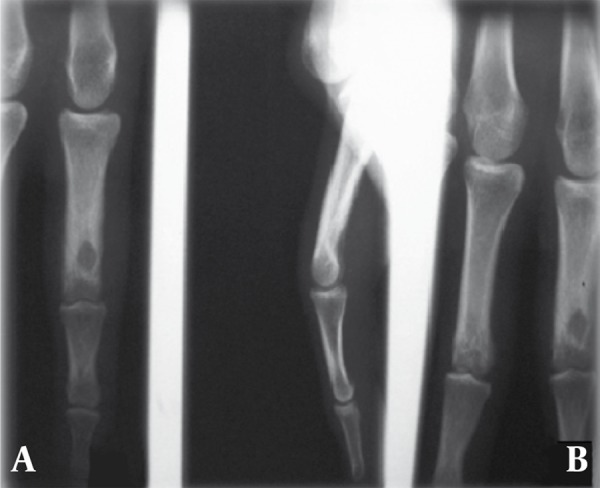
A 28-Year-Old Man With Pain in the Hand. A, Plain radiograghs show osteoid osteoma of the distal of the phalanx (medullary type) in AP and B, Lateral projection.

A 28-year-old man who had a history of tumor removal surgery about one year before presented with reswelling and relapse of the lesion. One year ago the tumor had involved the proximal phalanx of the second finger of the right hand. The pathologic evaluation had been compatible with osteoid osteoma. This time the patient complained of reswelling of the same place from 3 months before. The lesion relapsed in the form of an osteolytic lesion (medullary type) accompanied by tenderness and limitation of motion. The tumor resection confirmed relapse of the tumor (Figure 8).

### 2.3. Case 3

**Figure 9:**
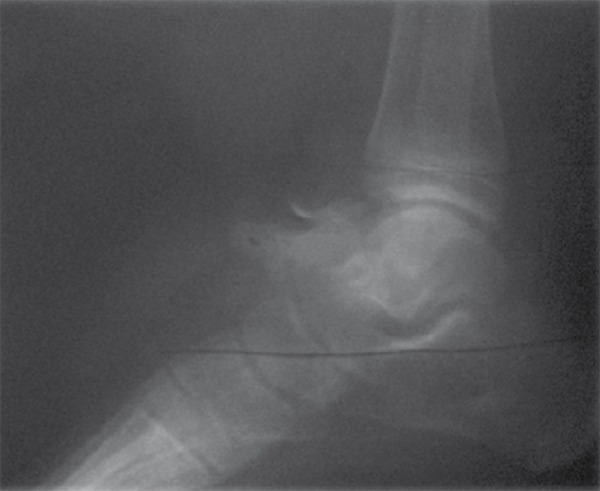
A 17-Year-Old Man Presenting With Foot Pain. Plain film shows subperiosteal osteoid osteoma of the talus which often presents as a round soft tissue mass adjacent to bone.

A 20-year-old man presented with a 2 year history of left ankle pain accompanied by swelling of the region. The pain occurred typically at night. Left ankle radiograph (lateral view) revealed features of subperiosteal osteoid osteoma along with a soft tissue mass around the lesion. (Figure 9).

### 2.4. Case 4

**Figure 10:**
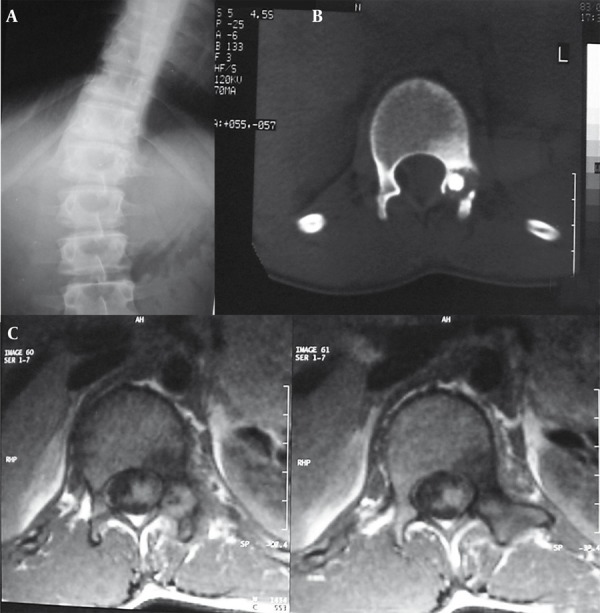
A 22-Year-Old Man With a Complaint of 5-Month Continuous Low Back Pain A, Radiograph revealed a sclerotic region in left pedicle of L1 vertebra along with scoliosis in thoracolumbar region. B, Lumbar CT scan revealed osteolytic and osteoblastic lesion in pedicle of L1 vertebra. C, MRI with T1 weighting demonstrated focal low signal intensity surrounded by soft tissue component replaced pedicle of L1 vertebra on the left side in the axial sections. Diffuse low signal intensity due to edema was seen in the adjacent bone.

A 22-year-old man presented with continuous low back pain for 5 months. The pain was not affected by motion and activity. Radiography revealed a sclerotic region in the left pedicle of the L1 vertebra along with scoliosis in the thoracolumbar region. CT scan also showed an osteolytic and osteoblastic lesion in the same place. Moreover, T1W MRI demonstrated focal low signal intensity surrounded by soft tissue component that replaced the pedicle of the L1 vertebra on the left side in the axial sections. Diffuse low signal intensity due to edema was seen in the adjacent bone. Radionuclide scan revealed an increased uptake in the same place (Figure 10). All the above findings were suggestive of both osteoid osteoma and osteoblastoma; though the surgical resection of the lesion confirmed the diagnosis of osteoid osteoma.

### 2.5. Case 5

A 9-year-old boy presented with a one-year pain in the right shin more dominant at night. In physical exam, a hard mass with bone consistency was detected in the mid part of the shin.

**Figure 11:**
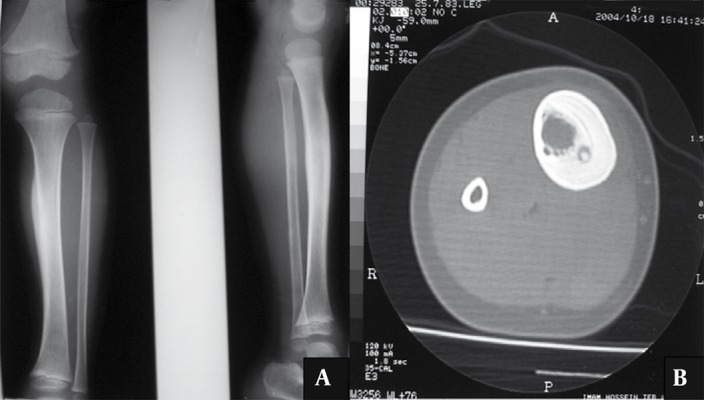
A Nine-Year-Old Boy With the Complaint of Right Shin Pain. A, The AP and lateral radiograph of the right shin revealed a solid periosteal reaction in the medial part of tibia without any obvious nidus. B, CT scan of the mid part of the right shin revealed a nidus along with a periosteal reaction around the lesion.

In radiography, a solid periosteal reaction in the medial part of the tibia without an obvious nidus was noticed. CT scan revealed a nidus along with a remarkable periosteal reaction around the lesion (Figure 11). Therefore, an osteoid osteoma may manifest as a periosteal reaction in plain radiography.

## 3. Discussion

We retrospectively studied the patients’ files with the pathological diagnosis of osteoid osteoma in the department of radiology at Imam Reza Hospital of Mashhad, University of Medical Sciences, Iran, within one year. Forty-four patients with osteoid osteoma were included. Plain films, CT images and isotope scans were available for all patients. Only five of these cases had also undergone MRI.

### 3.1. Clinical Symptoms

**Figure 12:**
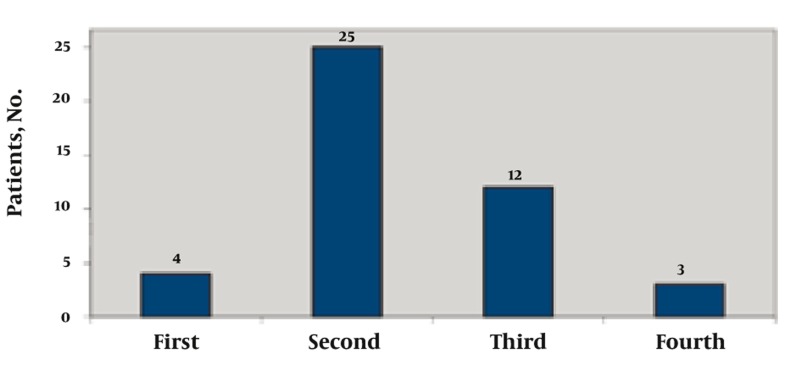
Distribution of Patients Based on Age Decade at Presentation

In this case series, there were 31 male and 14 female patients, making the diagnosis twice as common in men as in women. The mean age of the patients with osteoid osteoma was 17 years (range, 8-35 years). The distribution of the disease in different decades of life is shown in Figure 12. The second decade had the highest prevalence with 25 patients.

In most cases, radiological manifestations were seen simultaneously with clinical symptoms. In others, the pain preceded radiographic signs.

Pain was the only symptom in these patients which is initially mild and intermittent, but later becomes persistent and severe. The pain occurs generally at night. Only in one patient night pain was not reported. Favorable response to aspirin and NSAID was seen in 41 cases and only three of the patients showed a relative response. Ten patients noted radiating referral pain.

Additional clinical symptoms were swelling over the affected area seen in clinical examinations of nine patients and restricted movement in five. Pain increased with activity in six patients, while it was relieved by activity in 38 cases (Table 1).

**Table 1:** Clinical Symptoms

**Clinical Symptoms**	**No.**
Pain relieved by activity	38
Pain increased by activity	6
Lesion associated pain	43
Restricted movement of the affected limb	5
Referral pain	10
Swelling over the affected area	9

**Figure 13:**
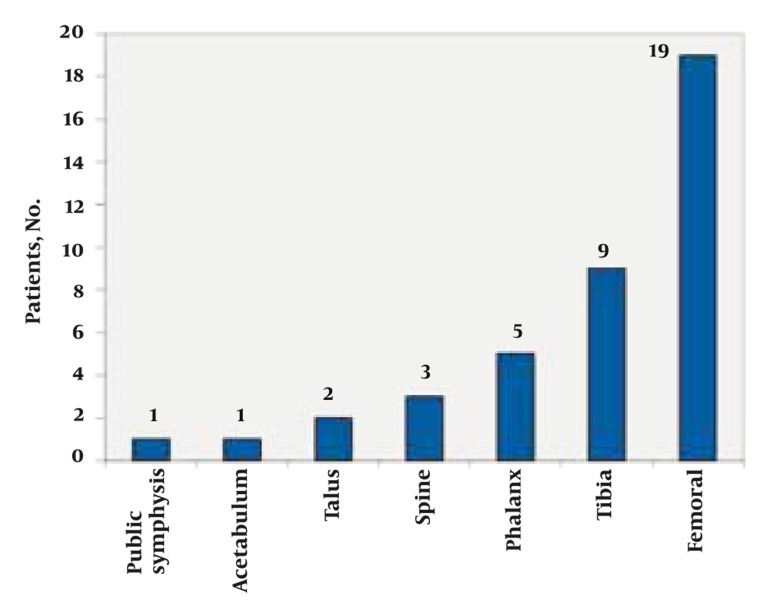
Osteoid Osteoma, Anatomical Sites of Origin Among 44 Patients

The average duration of pain reported by patients was approximately 17 months, within a range of 3 weeks to7 years from the onset of the disease. Recurrence of the disease after surgical resection was diagnosed in three patients in the same region. In one case the lesion was initially diagnosed in the femur, but after resection a second lesion was discovered in the pubic symphysis. Regarding the anatomic location of the tumor, the femoral neck and proximal tibia were commonly involved with 19 and 9 cases, respectively. Other affected sites, in order of prevalence included the humerus, talus, spine, phalanx, acetabulum and pubic symphysis (Figure 13).

### 3.2. Radiological Features

3.2.1. Plain Film

**Figure 14:**
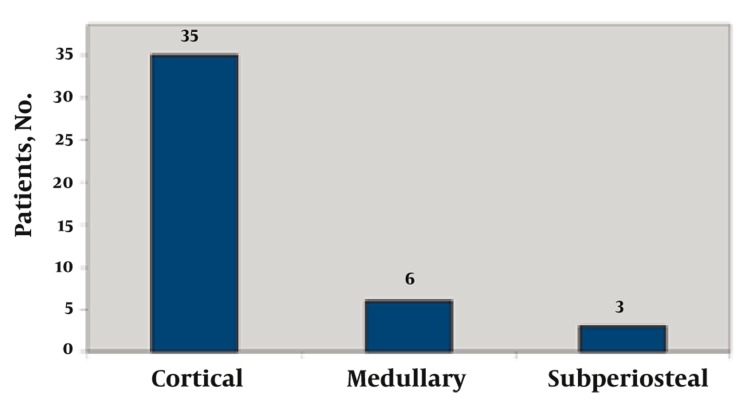
Distribution of Disease According to the Site of Origin

In our series, 35 patients presented with the cortical type, six cases were located in the medullary zone and three had subperiosteal osteoid osteoma (Figure 14).

3.2.2. CT Scan

In all the cases, the nidus was visualized. In one case where the lesion was located at T8 vertebra, CT scan only showed an increased density in the vertebra pedicle with a suspected nidus. Calcification and ossification within the nidus was either mild or absent in 15 cases. In two cases where the osteoid osteoma was located within the vertebra, scoliosis was present and the lesion was visualized as an increase of density in the pedicle (one thoracic and one lumbar vertebra). In the case with L1 lesion, increased density was seen in the body as well as the pedicle of the vertebra.

3.2.3. MRI

The nidus was visible in four out of five patients, who had also undergone MRI. In the fifth patient the nidus was only visible on CT. However, in all five cases signal changes related to bone marrow and soft tissue edema adjacent to the lesion were clearly detected in MRI.

3.2.4. Radionuclide Bone Scan

**Figure 15:**
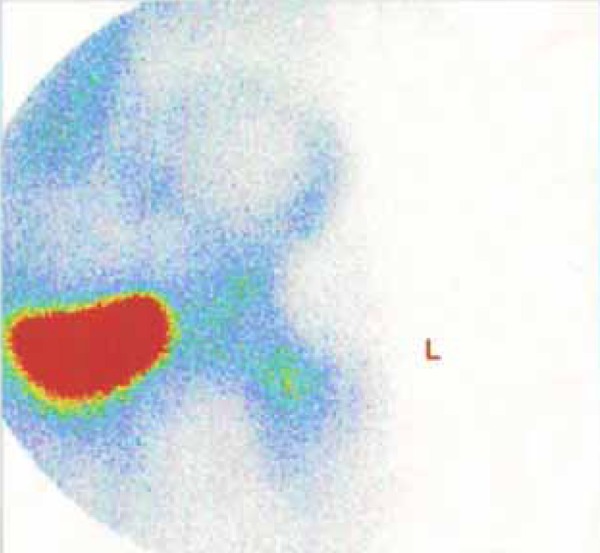
Double-Density Sign in the Femoral Neck

We used this sign together with a pathological report to verify the diagnosis of osteoid osteoma which was positive in all patients (Figure 15).

Finally, osteoid osteoma is known as a benign bone tumor, twice as common in males as in females. The highest incidence occurs in the second decade of life. Night pain relieved by aspirin is the most common symptom. Concerning the site of origin there are three types of osteoid osteoma; namely, cortical (classic type), medullary and subperiosteal.

The most common sites of involvement are the femoral neck and proximal tibia. This bone disease is detectable by plain film, CT, MRI and radionuclide bone scan, each bearing different characteristics in the diagnosis of this lesion. MRI is more sensitive in the diagnosis of bone marrow and soft tissue abnormalities adjacent to the lesion. The closer the nidus is to the medullary zone, the greater the role of MRI in detecting the nidus compared to CT. On the other hand, CT is more specific when it comes to detecting the lesion’s nidus.
